# Genomic deletion of chromosome 12p is an independent prognostic marker in prostate cancer

**DOI:** 10.18632/oncotarget.4626

**Published:** 2015-07-21

**Authors:** Martina Kluth, Ramin Ahrary, Claudia Hube-Magg, Malik Ahmed, Heinke Volta, Catina Schwemin, Stefan Steurer, Corinna Wittmer, Waldemar Wilczak, Eike Burandt, Till Krech, Meike Adam, Uwe Michl, Hans Heinzer, Georg Salomon, Markus Graefen, Christina Koop, Sarah Minner, Ronald Simon, Guido Sauter, Thorsten Schlomm

**Affiliations:** ^1^ Institute of Pathology, University Medical Center Hamburg-Eppendorf, Germany; ^2^ Martini-Klinik, Prostate Cancer Center, University Medical Center Hamburg-Eppendorf, Germany; ^3^ Dept. of Urology, Section for translational prostate cancer research, University Medical Center Hamburg-Eppendorf, Germany

**Keywords:** 12p deletion, prostate cancer, *CDKN1B*, p27, prognostic marker

## Abstract

Deletion of 12p is a recurrent alteration in prostate cancer, but the prevalence and clinical consequences of this alteration have not been studied in detail. Dual labeling fluorescence *in situ* hybridization using probes for 12p13 (*CDKN1B*; p27) and centromere 12 as a reference was used to successfully analyze more than 3700 prostate cancers with clinical follow-up data assembled in a tissue microarray format. *CDKN1B* was selected as a probe because it is located in the center of the deletion, which spans > 10 Mb and includes > 50 genes in 80% of cancers with 12p deletion. Deletion of 12p was found in 13.7% of cancers and included 13.5% heterozygous and 0.2% homozygous deletions. 12p deletion were linked to advanced tumor stage (*p* < 0.0001), high Gleason grade (*p* < 0.0001), rapid tumor cell proliferation (*p* < 0.0001), lymph node metastasis (*p* = 0.0004), and biochemical recurrence (*p* = 0.0027). Multivariate analysis including pT stage (*p* < 0.0001), Gleason grade (*p* < 0.0001), pN status (*p* = 0.0001), preoperative PSA levels (*p* = 0.0001), and resection margin status (*p* = 0.0001) revealed an independent prognostic value of 12p deletion (*p* = 0.0014). Deletion of 12p was unrelated to the ERG fusion status. Deletion of 12p was only marginally linked to reduced p27 expression, which by itself was unrelated to clinical outcome. This argues against p27 as the key target gene of 12p deletions. In summary, the results of our study demonstrate that 12p deletion is frequent in prostate cancer and provides independent prognostic information. 12p deletion analysis alone, or in combination with other prognostic parameters may thus have clinical utility.

## INTRODUCTION

Prostate cancer is the most frequent cancer in males. About 900,000 men are diagnosed with this disease every year, and almost 130,000 eventually die from their cancer in Western societies alone [1]. Autopsy studies suggest that more than 50% of males will develop prostate cancer during their lifetime, but only a minority of affected men will develop life-threatening disease that requires radical treatment [2, 3]. As screening strategies identify prostate cancers already at early stages of the disease, it becomes increasingly important to avoid overtreatment of patients with less aggressive disease. Establishing molecular markers enabling a distinction between indolent and aggressive forms of the prostate cancer is thus of utmost importance.

Chromosomal rearrangements are of considerable interest as diagnostic or prognostic biomarkers amongst others because they can reproducibly be tested due to their dichotomous nature being either present or not. Structural chromosomal rearrangements represent a major mechanism for activating and inactivating oncogenes and tumor suppressor genes in cancer. In contrast to most other malignancies, chromosomal rearrangements only rarely involve copy number gains or amplifications in prostate cancer. With the exception of the *TMPRSS2*:*ERG* fusion affecting about 50% of prostate cancers, all other individual translocations also occur at very low frequency (<5%) [4–6]. Many chromosomal deletions, however, are highly recurrent and occur in > 10% of cancers. The most common deletions include 8p (40–50%), 13q14, 16q22-q24, 6q12-q22, 10q23 (20–30% each), 12p12-p13, 3p13 (15–20% each), and 5q21 (10%) [6–10]. It is not fully understood, how these deletions impact prostate cancer cells and their exact mechanisms of action may vary between deleted loci. Very small deletions may impact one specific gene. For example deletions at 10q23 are typically narrow and are likely to specifically target *PTEN*, a gene with tumor suppressive properties, which has been widely studied in prostate cancer [6, 8, 11]. Most other deletions are substantially larger and may include hundreds of genes [6–10]. Even though some of these deletions contain known tumor suppressor genes, such as the retinoblastoma gene on 13q14, it remains doubtful that the loss of large pieces of DNA just serves the purpose of affecting just one single gene. The one deletion/one gene hypothesis is also disturbed by the adamant lack of inactivating mutations seen in the retained allele of the best candidate tumor suppressor genes in studies utilizing next generation sequencing [4, 5, 12, 13].

Irrespective of their impact on gene function, several deletions have shown a striking prognostic impact in prostate cancer. This applies for example for deletions of *PTEN* [11], 17p13 [14], 5q21 [15], 6q15 [16], and 8p [17]. Other frequent deletions, such as deletions of 12p, have so far not been analyzed for their potential prognostic role. In order to study the prognostic impact of 12p copy number alterations, we analyzed more than 7,000 prostate cancers with clinical follow-up data by fluorescence *in situ* hybridization (FISH). The results of our study identify 12p deletion as a strong independent molecular prognostic feature in prostate cancer.

## RESULTS

### Technical aspects

12p FISH analysis was successful in 3,757 of 7,482 (50.2%) arrayed cancers. Analysis was not informative in the remaining 3,725 tumors because of lack of tumor cells in the tissue spots, faint or absent FISH signals, or missing tissue spots on the TMA section. The distribution of clinical and pathological parameters in the 3,757 cancers with interpretable FISH results and the 3,725 cancers without interpretable FISH results was comparable.

### 12p deletions and prostate cancer phenotype

12p deletions were found in 13.7% (514 of 3,757) of all prostate cancers, including 13.5% heterozygous and 0.2% homozygous deletions. The relationship between 12p deletions and tumor phenotype and clinical parameters is summarized in Table 1. 12p deletions were significantly linked to high Gleason grade (*p* < 0.0001), advanced tumor stage (*p* < 0.0001), presence of lymph node metastasis (*p* = 0.0004), and elevated preoperative PSA values (*p* = 0.0027).

**Table 1:** Associations between 12p deletion and prostate cancer phenotype in all, ERG fusion positive, and ERG fusion negative tumors

	All cancers			ERG-negative cancers			ERG-positive cancers		
	*n*	12p deletion (%)	*p* value	*n*	12p deletion (%)	*p* value	*n*	12p deletion (%)	*p* value
	3757	13.7%		1821	14.4%		1678	13.7%	
**Tumor stage**									
pT2	2312	11.4%	<0.0001	1176	12.2%	<0.0001	960	11.8%	0.0630
pT3a	911	15.5%		397	13.9%		468	16.9%	
pT3b	468	20.5%		219	26.5%		218	14.7%	
pT4	42	21.4%		21	23.8%		17	17.7%	
**Gleason grade**									
≤ 3 + 3	1135	8.6%	<0.0001	547	7.1%	<0.0001	486	10.7%	0.0038
3 + 4	1853	14.5%		861	16.0%		881	13.6%	
4 + 3	583	18.5%		303	21.1%		244	16.4%	
≥ 4 + 4	161	21.7%		101	19.8%		52	28.9%	
**Lymph node metastasis**									
N0	2160	15.1%	0.0004	1049	15.4%	0.0023	987	15.3%	0.0743
N+	173	26.1%		82	29.3%		82	23.2%	
**PSA Level (ng/μl)**									
<4	466	10.1%	0.0027	201	11.9%	0.0909	226	8.4%	0.0206
4–10	2128	13.1%		1014	13.4%		967	13.4%	
10–20	804	15.6%		438	16.2%		319	16.0%	
>20	294	18.7%		144	20.1%		130	18.5%	
**Surgical margin**									
negative	2894	13.1%	0.1224	1432	13.8%	0.1831	1251	13.1%	0.6165
positive	796	15.2%		361	16.6%		390	14.1%	

### 12p deletion and ERG fusion status

**Figure 1:**
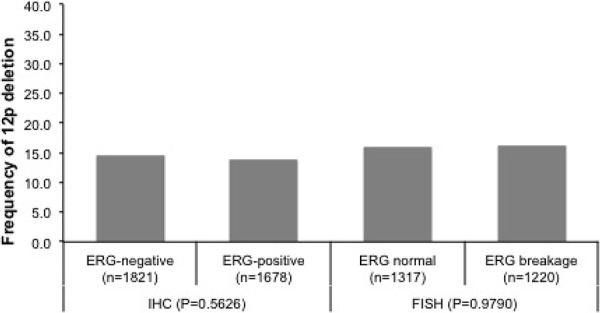
Relationship between 12p deletion and ERG fusion probed by IHC and FISH

12p deletions were unrelated to ERG fusion status irrespective from the method of ERG analysis (*p* = 0.5626 for ERG-IHC and *p* = 0.9790 for *ERG*-FISH analysis). Deletions of 12p were found in 14.4% and 16.0% of ERG-negative cancers (according to ERG IHC and FISH analysis), and in 13.7% (IHC, *p* = 0.5626) and 16.0% (FISH, *p* = 0.9790) of ERG-positive cancers (Figure 1). There was no major difference in the relationship between 12p deletions and tumor phenotype between ERG-positive and ERG-negative cancers. Most associations of 12p deletions and tumor phenotype parameters held also true in subgroup analyses (Table 1).

### 12p deletion and p27 expression

p27 expression data were available from 2,125 patients for whom 12p deletion data were also available. p27 was negative in 16.6%, weak in 34.8%, moderate in 29.4%, and strong in 19.2% of these cases. Loss of p27 immunostaining was linked to tumors of low Gleason grade (*P* < 0.0001) and ERG fusion negative cancers (*P* < 0.0001). Reduced (negative or weak) p27 expression was found in 59.7% of 12p deleted and in 50.4% of 12p undeleted cancers (*p* = 0.0080; Table 2).

**Table 2:** Associations between p27 expression and Gleason grade, ERG fusion status, and 12p deletion

	p27 status in all cancers					
	*n*	negative	weak	moderate	strong	*p* value
**Gleason grade**						
≤ 3 + 3	1305	25.13	32.11	26.28	16.48	<0.0001
3 + 4	1755	15.16	34.64	29.23	20.97	
4 + 3	444	15.09	33.78	30.18	20.95	
≥ 4 + 4	145	8.28	33.1	34.48	24.14	
**ERG fusion status**						
negativ	1602	27.53	38.45	22.28	11.74	<0.0001
positive	1536	6.9	30.92	35.68	26.5	
**12p deletion status**						
normal	1892	17.6	42.06	28.33	28.33	0.0075
deleted	233	16.49	33.93	29.49	20.08	

### 12p deletions and clinical outcome

**Figure 2:**
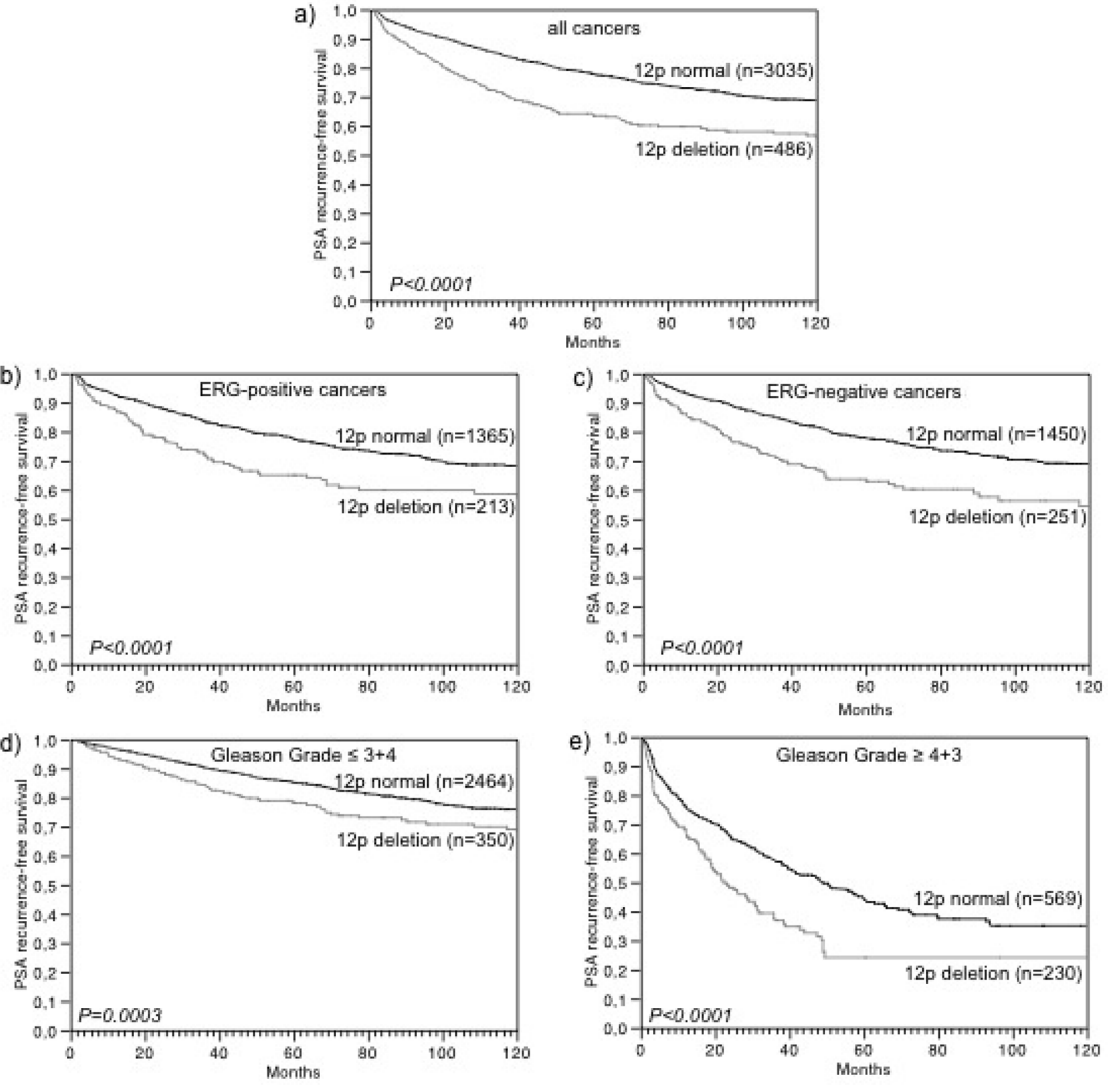
Association of 12p deletion with biochemical recurrence in **a.** all prostate cancers, **b.** ERG-positive prostate cancers, **c.** ERG-negative prostate cancers, **d.** low-grade cancers, and **e.** high-grade cancers.

**Figure 3:**
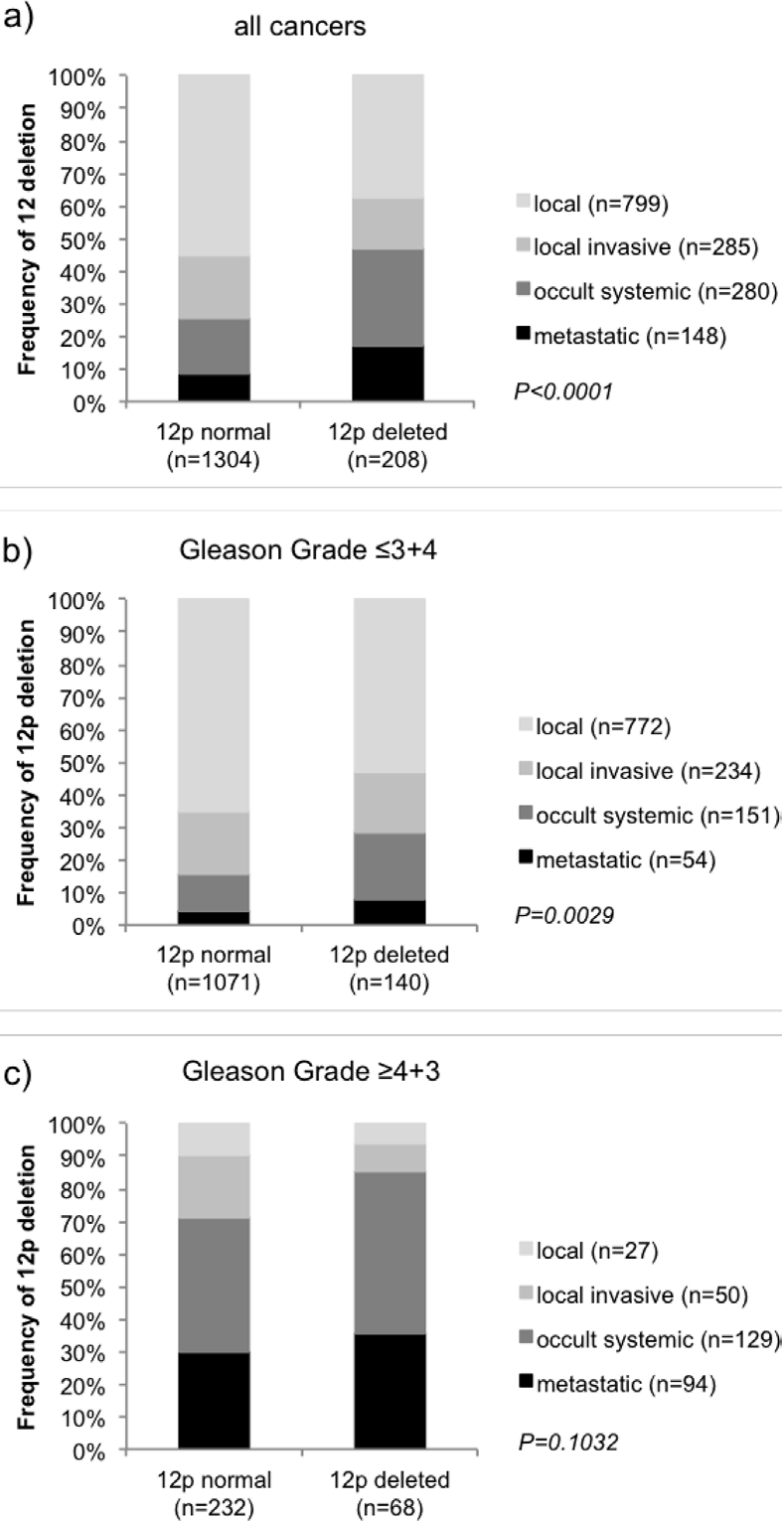
Association of 12p deletion with clinical groups representing the clinical hallmarks of prostate cancer in **a.** all tumors and subsets of **b.** low-grade and **c.** high-grade cancers.

Follow-up data were available from 3,521 tumors that were successfully analyzed for 12p deletion. In univariate analysis, 12p deletions were strongly linked to early biochemical (PSA) recurrence in all cancers (*p* < 0.0001, Figure 2a) and there was no difference seen in the prognostic impact of 12p deletions 1, 701 ERG-negative (*p* < 0.0001, Figure 2b) and 1,578 ERG-positive cancers (*p* < 0.0001, Figure 2c). Significant associations with PSA recurrence held also true in subsets of cancers with low (Gleason ≤ 3 + 4, *p* = 0.0003) or high Gleason (≥ 4 + 3, *p* < 0.0001, Figure 2d–2e). 12p deletion status was also significantly associated to unfavorable outcome according to our alternative clinical endpoints, which represent the four hallmarks (local, local invasive, occult systemic and metastatic) of tumor growth and dissemination in prostate cancer (Figure 3). More than 70% of patients without 12p deletion had either local or local invasive disease while this fraction decreased to 53% in patients with 12p deletion (*p* < 0.0001, Figure 3a). A separate analysis of low grade (Gleason ≤ 3 + 4 = 7) and high grade (Gleason ≥ 4 + 3 = 7) cancers revealed that the prognostic impact of 12p deletions was particularly strong in low-grade cancers (*p* = 0.0029) while it largely disappeared in high grade tumors (*p* = 0.1032, Figure 3b–3c). In a multivariate analysis including the established prognostic predictors pT stage, Gleason grade, nodal stage, resection margin status and preoperative PSA level, 12p deletion predicted early PSA recurrence independently from these parameters in all cancers (*p* = 0.0014) and in the subset of ERG-positive cancers (*p* = 0.0022). It only marginally fell short of reaching statistical significance in ERG-negative cancers *p* = 0.0723, Table 3).

**Table 3:** Multivariate analysis including established prognostic parameters and 12p deletion in all, ERG fusion positive, and ERG fusion negative tumors

	all prostate cancers			ERG-negative prostate cancers			ERG-positive prostate cancers		
Parameter	RR	95% CI	*p*-value	RR	95% CI	*p*-value	RR	95% CI	*p*-value
**Tumor stage**									
pT3a vs pT2	2.0	1.6–2.5	<0.0001	1.8	1.4–2.4	<0.0001	2.1	1.5–2.8	*<0.0001*
pT3b vs pT3a	1.6	1.3–1.9		1.3	1.0–1.8		2.1	1.6–2.7	
pT4 vs pT3b	1.8	1.2–2.5		1.9	1.1–3.1		1.1	0.6–1.9	
**Gleason grade**									
3 + 4 vs ≤ 3 + 3	2.0	1.5–2.6	<0.0001	1.5	1.1–2.3	<0.0001	2.9	1.9–4.6	*<0.0001*
4 + 3 vs 3 + 4	2.1	1.8–2.5		2.0	1.6–2.6		2.1	1.6–2.7	
≥ 4 + 4 vs 4 + 3	1.0	0.8–1.3		1.0	0.7–1.4		1.2	0.8–1.8	
**Nodal stage**									
pN1 vs pN0	1.6	1.3–2.0	0.0001	1.8	1.2–2.5	0.0024	1.3	0.9–1.8	*0.1351*
**Resection margin status**									
R1 vs R0	1.4	1.2–1.6	0.0001	1.5	1.2–1.9	0.0010	1.3	1.0–1.6	*0.0340*
**Pre-operative PSA (ng/ml)**									
4–10 vs <4	1.0	0.7–1.3	<0.0001	1.0	0.6–1.6	0.0005	0.9	0.6–1.4	*0.1668*
10–20 vs 4–10	1.3	1.1–1.5		1.5	1.2–1.9		1.1	0.9–1.4	
>20 vs 10–20	1.3	1.0–1.6		1.2	0.9–1.6		1.3	0.9–1.7	
**12p status**									
12p deletion vs normal	1.4	1.1–1.6	0.0014	1.3	1.0–1.7	0.0723	1.6	1.2–2.1	*0.0022*

### 12p deletion and cell proliferation

12p deletions were linked to increased cell proliferation as measured by Ki67 immunohistochemistry. This was true for all tumors but also for most subgroups of Gleason ≤ 3 + 3 = 6, 3 + 4 = 7, 4 + 3 = 7, and ≥ 4 + 4 = 8 (Table 4).

**Table 4:** Association between cell proliferation as measured by Ki67LI immunohistochemistry and 12p deletion in tumor subsets of different Gleason grade

	12p status	n evaluable	Ki67 Li mean	Std.deviation
all cancers *P* < 0.0001	normal	2220	2.67	0.05
	deleted	385	3.31	0.13
pGleason ≤ 3 + 3 *P* = 0.0172	normal	696	2.22	0.08
	deleted	75	2.85	0.25
pGleason 3 + 4 *P* = 0.0017	normal	1118	2.67	0.07
	deleted	202	3.20	0.16
pGleason 4 + 3 *P* = 0.8961	normal	311	3.26	0.18
	deleted	81	3.31	0.35
pGleason ≥ 4 + 4 *P* = 0.1025	normal	81	4.10	0.48
	deleted	24	5.75	0.88

### Location and extension of 12p deletions in prostate cancer

**Figure 4:**
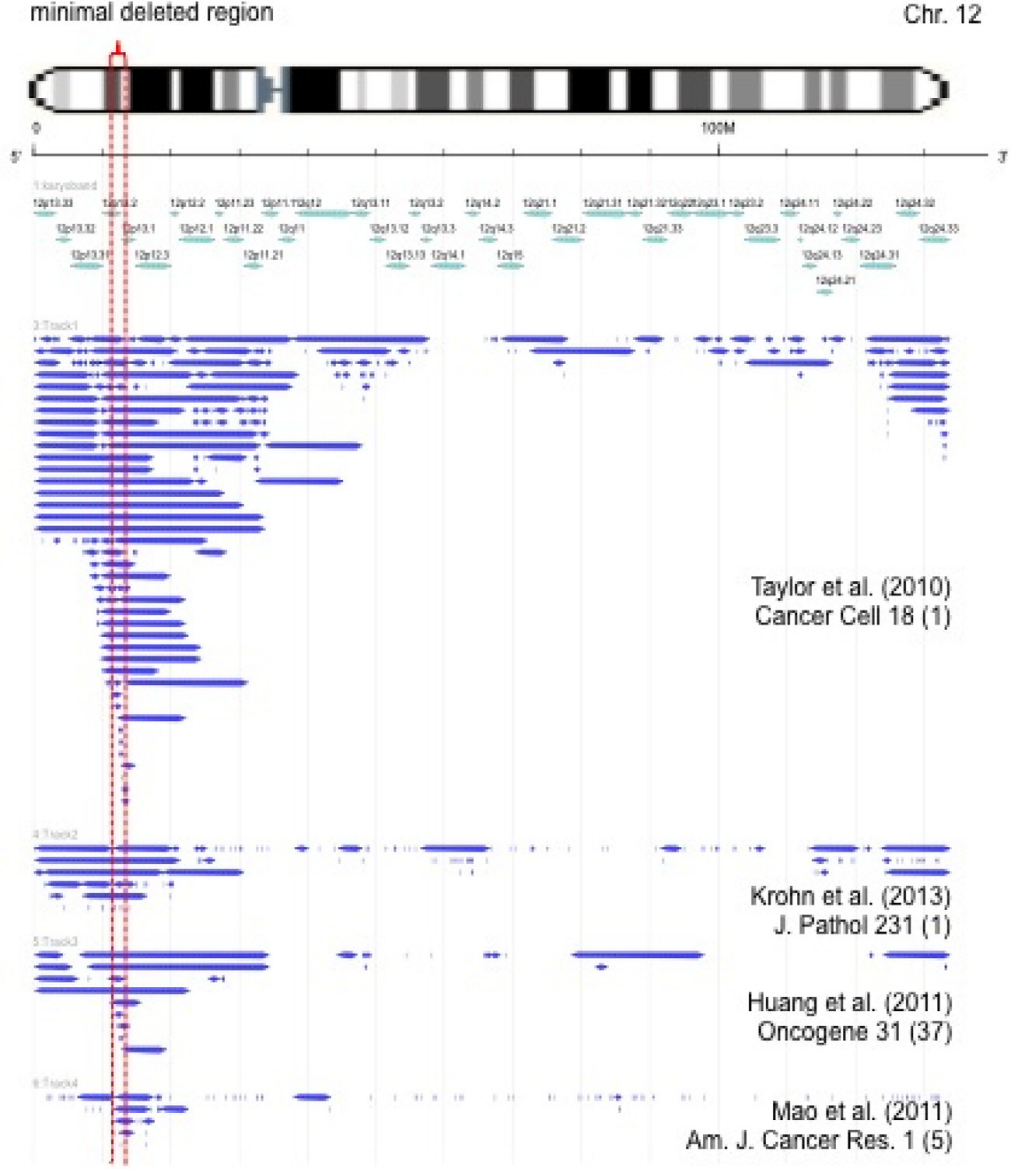
Size and extension of chromosome 12p deletions detected in published microarray-based copy number studies [6, 55–59] Each bar represents the deleted area in a single tumor. The minimal commonly deleted region is indicated be red dotted lines.

Data on the patterns of 12p deletions from own previous analyses and from the literature are summarized in Figure 4. The data demonstrate that >80% of 12p deletions are larger than 10 Megabases (Mb) and contain more than 50 genes. The minimal common deletion is at 12p13.1, spans about 1.5 Mb and contains 7 genes including *DUSP16*, *CREBL2*, *GPR19*, *CDKN1B*, *APOLD1*, *DDX47*, and *GPRC5A*.

## DISCUSSION

The results of our study demonstrate that 12p deletion occurs in a relevant fraction of prostate cancers and has a strong and independent prognostic relevance in this tumor.

About 14% of more than 3,500 prostate cancers successfully analyzed by FISH showed 12p deletions in our study. Earlier studies employing classical or array-based comparative genomic hybridization or loss of heterozygosity (LOH) analysis have often found higher rates. Classical CGH studies have described 12p deletions in 17% of 6 [18] and 12% of 52 [19] cancers. Array based CGH analyses have found 12p deletions in 24% of 46 [20], 22% of 48 [21], 27% of 64 [8] and 12% of 181 [6] prostate cancers. The somewhat higher rate of 12p deletions in many of these studies may partly be due to a bias caused by small patient numbers or patient selection, as several projects focused on advanced, hormone refractory or metastatic cancers [21–24]. The highest rates of 12p deletions described in prostate cancer are derived from PCR-based loss of heterozygosity (LOH) studies. Here, authors described 12p LOH in 12% of 17 [24], 23% of 99 [22] and 49% of 19 cancers [23]. The high rates of LOH found in some studies may be due to some false positive cases caused by chromosome 12 polysomy in non-diploid tumors that account for 30–50% of prostate cancers [25, 26]. FISH is regarded as the gold standard for gene copy number determination as it allows for analysis on the single cell level, and is not disturbed by contaminating non-neoplastic cells that are inevitably present in cancer tissues. While other FISH studies dealing with 12p deletions in prostate cancer are lacking, we are confident with our data, also because the selected threshold of 60% of cells required to have fewer 12p than centromere 12 signals to call a “12p deletion” has been earlier extensively validated by our group for *PTEN* deletions [11].

Deletions of 12p were significantly associated with advanced tumor stage, high Gleason grade, high preoperative PSA value, lymph node metastasis, accelerated tumor cell proliferation, and early biochemical recurrence. These findings are in line with earlier data, which already suggested a possible link of 12p deletions with unfavorable tumor phenotype. Taylor et al. described a significant association of 12p deletion with Gleason grade and pT stage in a series of 181 cancers [6]. The mechanism causing increased aggressiveness of cancer cells with 12p deletion is not clear. Own earlier data and data from the literature demonstrate, that the deleted area typically spans more than 25 Mb (Figure 4) and contains more than 100 genes. We selected a FISH probe targeting the *CDKN1B* locus (Cyclin-Dependent Kinase Inhibitor 1B, 12p13.1) because *CDKN1B* is located in the center of the commonly deleted 12p region [6], and it encodes p27/kip1, a well known tumor suppressor gene with importance in various cancer types (reviewed in [27, 28]. Several studies have utilized IHC to analyze p27 in prostate cancer and reported a reduced p27 expression in 12.5%–84.3% of tumors. Several authors described that reduced p27 expression was linked to unfavorable tumor phenotype and prognosis [29–38] but others could not confirm these observations [33, 34, 39–43]. In an own study on 4,699 carcinomas, we also failed to see a prognostic impact of reduced p27 expression [44]. Altogether the published data argue against a strong prognostic impact of reduced p27 expression in prostate cancer. The only marginal association of reduced p27 expression with 12p deletion in this study further argues against p27 playing a critical role for increased aggressiveness of 12p deleted cancer cells.

The one deletion/one gene hypothesis is currently challenged by the adamant lack of inactivating mutations seen in the retained allele of the best candidate tumor suppressor genes in studies utilizing next generation sequencing [4, 5]. In fact, the most recurrently mutated genes are located outside the typical deletion regions (e.g. *SPOP* at 17q21, *FOXA1* at 14q12, or *ZNF595* at 4p16), and for the common large deletions such as 8p, 5q, 6q, 16q, and 13q, recurrent mutation have not been found [4–6, 12, 13]. These findings challenge the classical recessive model of biallelic tumor suppressor gene inactivation. The biological mechanism driving the development of large deletions involving hundreds of genes is not known. It is tempting to speculate, however, that simultaneous dosage reduction of multiple genes within such a deletion jointly support tumor growth. Such cooperative effects have indeed reported from mouse orthologs of human 8p11-p23 genes in a mouse model of HCC [45]. Of note, large 8p deletion, often involving the entire chromosome arm, is one of the most frequent alterations in many solid tumor types including prostate cancer and attempts to identify “the critical 8p gene” have so far failed [6–8]. Beyond the cell cycle regulator *CDKN1B* (12p13.1), further potentially relevant cancer genes on 12p may for example include *CD9*, *ING4*, and *BCL-G*. *CD9* (12p13.31) regulates multiple cellular functions such as cell adhesion, migration, apoptosis, and tumor cell motility [46], *ING4* (12p13.31) is involved in cell cycle arrest, apoptosis and senescence [47], and *BCL-G* (12p13.2) plays a role in apoptosis [47].

Chromosomal deletions analyzed in large prostate cancer cohorts have so far shown striking associations with either ERG-positive or ERG-negative cancers. For example, deletions of 6q15 and 5q21 are frequent in ERG fusion negative cancers, whereas deletions of 3p13, *TP53* and *PTEN* are common in ERG fusion positive cancers. It has been suggested, that ERG induced “reprogramming” of the cellular environment can facilitate or inhibit the development of certain deletions or vice versa, and that certain deletions may induce gene expression changes that inhibit the development of *ERG* rearrangements [4–6, 48]. Deletions that are independent of the ERG status have so far not been described. The independence of 12p deletions of *ERG* rearrangements is thus remarkable and might suggest, that either different – ERG independent – mechanisms may apply for 12p deletion development or that – in contrast to most other recurrent deletions - none of the affected genes is influencing possible mechanisms needed for the development of *TMPRSS2*:*ERG* fusion.

The strong link of 12p deletions with the probability for PSA recurrence is of potential clinical relevance especially since this was independent of established clinical and pathological parameters. However, the selection of clinical endpoints is not trivial in studies evaluating prostate cancer. It is obvious, that PSA recurrence does not represent an optimal clinical endpoint, even though it is used in the vast majority of studies. More than 30% of prostate cancer patients experience a PSA recurrence after surgical therapy within ten years, but only a minority of these cancers (<10%) will eventually progress to life threatening disease [49]. Moreover, PSA recurrence barely represents the natural history of prostate cancer after radical prostatectomy. Even cancer specific death – often considered as an optimal clinical endpoint - is suboptimal in prostatectomy cohorts because knowing the risk to die from one’s cancer despite maximal multimodal therapy concepts - including radical surgery, adjuvant radiation and/or hormonal- and cytotoxic therapy - has limited practical value in the absence of established additional adjuvant treatment options.

In an attempt to define clinical endpoints that could better support pre-therapeutic decision making at the time of the initial cancer diagnosis we defined clinical groups, representing the two clinical hallmarks of cancer, i.e., local tumor extension and systemic tumor growth and that can be analyzed on materials derived from prostatectomy specimen. These clinical groups were selected to reflect the “biological milestones” of cancer progression, including 1) organ-confined tumors (pT2) without relapse in long-term follow-up – and thus assumed “local”, 2) “local invasive” cancers (pT3) without relapse or with permanent response to local secondary radiation in long-term follow-up, 3) “occult systemic” disease characterized by failure of two local therapies (radical prostatectomy and secondary radiation), but no evidence of distant metastases in long-term follow-up, and 4) “metastatic” disease characterized by regional or distant metastases. That this analysis also revealed a strikingly increased risk for presence of occult systemic or manifest metastatic disease in 12p deleted further supports the potential clinical significance of this deletion.

In summary, these data identify 12p deletion as a frequent event in prostate cancer, which is unrelated to the ERG fusion status but strongly linked to aggressive tumor behavior. The large average size of the deletion, the only marginal association with p27 protein expression and the lack of prognostic impact of p27 expression argues against p27 representing the sole or critical target gene. Irrespective of the target gene(s), 12p deletion analysis may have clinical utility for prognostic testing of prostate biopsies either alone or in combination with other features.

## MATERIALS AND METHODS

### Patients

A set of prostate cancer tissue microarrays (TMA) was used in this study containing one tissue core each from 7,482 consecutive radical prostatectomy specimens from patients undergoing surgery at the Department of Urology, and the Martini Clinic, Prostate Cancer Center, University Medical Center Hamburg-Eppendorf. This TMA is based on our previous 3,261 samples prostate prognosis TMA [17], with additional 4,634 tumors and updated clinical data from 6,894 patients with a median follow-up of 38.8 months (range: 1 to 241 months; Table 5). In all patients, prostate specific antigen (PSA) values were measured quarterly in the first year, followed by biannual measurements in the second and annual measurements after the third year following surgery. Different clinical endpoints were defined in the patient set: Time to recurrence was defined as the time interval between surgery and first occurrence of a postoperative PSA of ≥0.2 ng/ml and rising thereafter. Patients without evidence of tumor recurrence were censored at the time of the last follow-up. In addition, patients were grouped into subsets defining the milestones of tumor progression: Group 1 “organ confined tumor growth” included 1,458 patients with organ confined tumors (pT2) and no biochemical relapse (BCR) in long-term follow-up (at least 3y after surgery; mean 122.2 (120.5–129.9) months), Group 2 “local invasive tumor growth” included 446 patients with histological proof of extraprostatic tumor growth (pT3a or pT3b), but no BCR in long-term follow-up (at least 3y after surgery; mean 125.2 (121.2 – 129.2) months) or pT3 tumors with BCR but permanent response to secondary adjuvant or salvage radiation therapy, Group 3 “occult systemic dissemination” included 517 cancers with biochemical failure after two local therapies (radical prostatectomy and additional adjuvant or salvage- radiation therapy), and without development of distant metastases in long-term follow-up (mean follow-up 87.1 (83.3–91.0) months), and Group 4 “metastatic tumor growth” included 199 patients with development of distant (bone and/or visceral) or lymph node metastases (mean follow-up 95.5 (89.0–102.0) months). In group 1–3, patients with neoadjuvant or post operative hormonal therapies were excluded. The remaining 4 714 cancers could not be classified into one of these groups, mainly because clinical information was incomplete or the follow-up time was to short. All prostate specimens were histologically examined according to a standard procedure, including complete embedding of the entire prostate for histological analysis [50]. The TMA manufacturing process was described earlier in detail [51, 52]. In short, one 0.6 mm core was taken from a representative tissue block from each patient. The tissues were distributed among 16 TMA blocks, each containing 144 to 522 tumor samples. Presence or absence of cancer tissue was validated by immunohistochemical AMACR and 34BE12 analysis on adjacent TMA sections. Each TMA block also contained various control tissues, including normal prostate tissue. Analysis of patient and corresponding histopathological data for research purposes, as well as construction of tissue microarrays from archived diagnostic left-over tissues, was approved by local laws (HmbKHG, §12, 1) and by the local ethics committee (Ethics commission Hamburg, WF-049/09 and PV3652). All work was carried out in compliance with the Helsinki Declaration. The molecular database attached to this TMA includes data on ERG expression in 6,135, on *ERG* rearrangement by FISH analysis in 3,688 (extended from [53]), as well as immunohistochemical data on p27 expression from 3,701 [44] and KI67 labeling Index (Ki67 LI) data in 4,426 patients (extended from [54]).

**Table 5:** Clinico-pathological features of 7 482 arrayed prostate cancers

	No. of patients (%)	
	Study cohort on TMA (*n* = 7482)	Biochemical relapse among categories
**Follow-up (mo)**		
*n*	7482 (60.2%)	1457 (19.5%)
Mean	53.4	-
Median	36.8	-
Age (y)		
≤50	234 (3.2%)	43 (18.4%)
51–59	1912 (25.8%)	368 (19.2%)
60–69	4438 (59.9%)	872 (19.6%)
≥70	822 (11.1%)	172 (20.9%)
**Pretreatment PSA (ng/ml)**		
<4	976 (13.2%)	125 (12.8%)
4–10	4443 (60.3%)	650 (14.6%)
10–20	1461 (19.8%)	411 (28.1%)
>20	488 (6.6%)	248 (50.8%)
**pT category (AJCC 2002)**		
pT2	4927 (66.2%)	460 (9.3%)
pT3a	1650 (22.2%)	477 (28.9%)
pT3b	803 (10.8%)	472 (58.8%)
pT4	58 (0.8%)	48 (82.8%)
**Gleason grade**		
≤ 3 + 3	2316 (31.2%)	171 (7.4%)
3 + 4	3804 (51.2%)	693 (18.2%)
4 + 3	1018 (13.7%)	448 (44%)
≥ 4 + 4	287 (3.9%)	144 (50.2%)
**pN category**		
pN0	3963 (92.4%)	919 (23.2%)
pN+	328 (7.6%)	207 (63.1%)
**Surgical margin**		
Negative	5921 (80.6%)	914 (15.4%)
Positive	1428 (19.4%)	516 (36.1%)

NOTE: Numbers do not always add up to 7482 in the different categories because of cases with missing data. Abbreviation: AJCC, American Joint Committee on Cancer.

### Fluorescence *in-situ* hybridization

**Figure 5:**
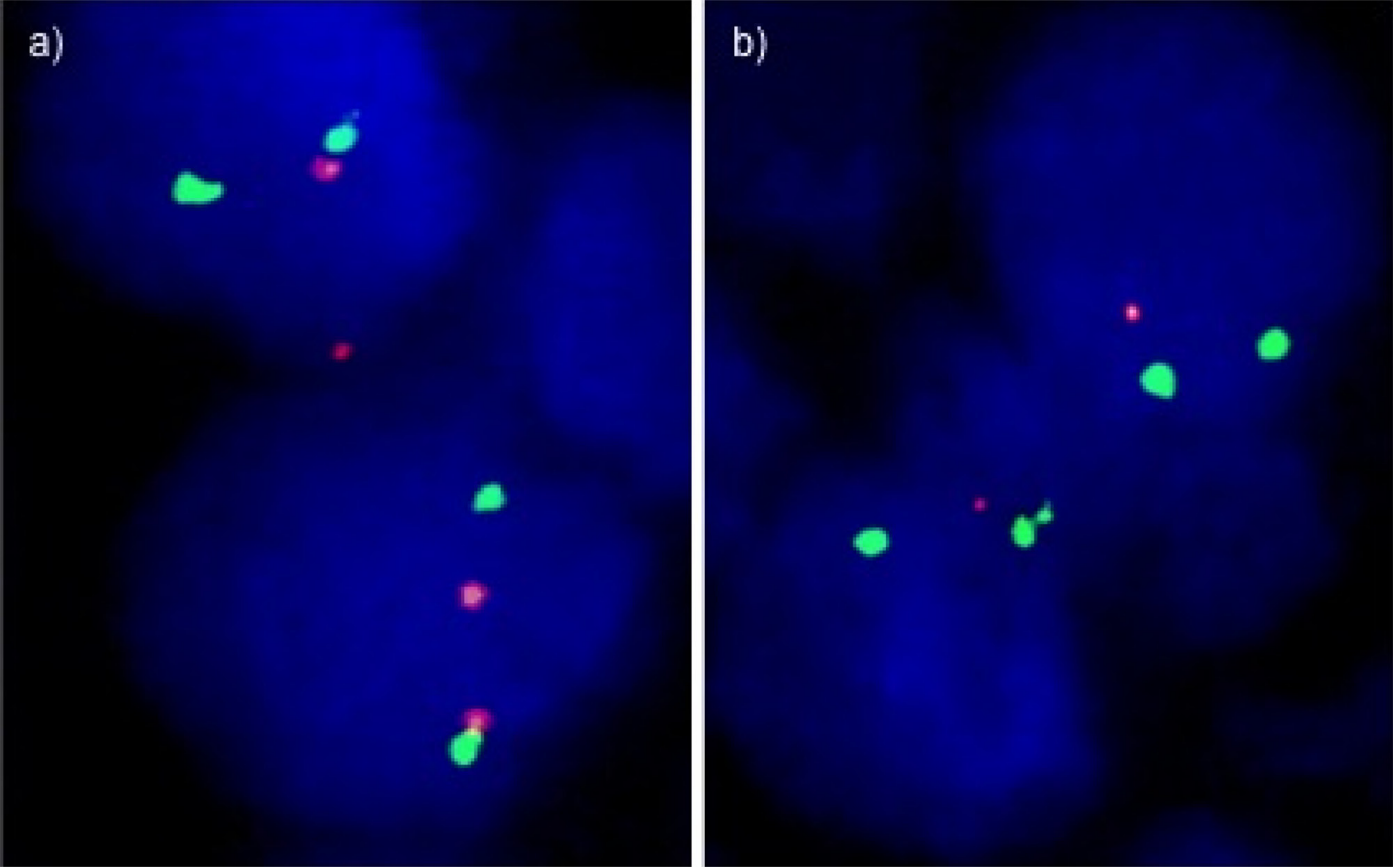
Examples of FISH findings showing **a.** normal 12p signal numbers and **b.** heterozygous 12p deletion. Green signals correspond to centromere 12, red signals correspond to the *CDKN1B* (12p13) gene locus.

Four micrometer TMA sections were used for fluorescence in-situ hybridization (FISH). TMA sections were de-waxed, air-dried, and dehydrated in 70%, 85%, and 100% ethanol. Slides were pretreated in VP 2000 Pretreatment Reagent (Abbott, Des Plaines, USA) for 15 min at 80°C, followed by 150 min incubation at 37°C in 0.5% protease 1 solution (Abbott, Des Plaines, USA). 4 μl of FISH probe mix in 70% formamide 2x SSC solution was applied to the slides and co-denatured with the cellular DNA in a Hybrite hybridization oven for 10 min at 72°C prior to overnight-hybridization at 37°C in a humidified chamber. The FISH probe mix consisted of a spectrum-orange labeled 12p (CDKN1B locus, 12p13.1) probe (made from BACs RP11–180M15 and BAC RP11–113I21), and a spectrum-green labeled, commercial centromere 12 probe (#6J37–12; Abbott, Wiesbaden, Germany) as a reference. After hybridization, slides were subjected to serial stringent washings (2x SSC solution with 0, 3% NP40 at 72°C for 2 minutes) and counterstained with 0.2 μmol/L 4′-6-diamidino-2-phenylindole (DAPI) in antifade solution. Stained slides were manually interpreted under an epifluorescence microscope, and the predominant green and orange FISH signal numbers were recorded in each tissue spot. Homozygous deletion of 12p was defined as complete lack of 12p FISH signals in the tumor nuclei, but presence of 12p FISH signals in adjacent normal cells. Tissue spots lacking 12p signals in all (tumor and normal cells), or lacking of any normal cells as an internal control for successful hybridization of the 12p probe, were excluded from analysis. Heterozygous deletion of 12p was defined as the presence of fewer 12p signals than centromere 12 probe signals of ≥ 60% tumor nuclei. These thresholds were based on a previous study analyzing PTEN deletions in a subset of slides of the TMA set [11]. Representative FISH images are shown in Figure 5.

### Statistics

For statistical analysis, the JMP software (SAS Institute Inc., NC, USA) was used. Contingency tables were calculated to study association between 12p deletion and clinico-pathological parameters, and the Chi-square (Likelihood) test was used to find significant relationships. Kaplan Meier plots were generated for PSA recurrence-free survival. The Log-Rank test was applied to determine the significance of differences between the survival curves. Cox proportional hazards regression analysis was performed to test the statistical independence and significance between pathological, molecular, and clinical variables.
