# Correction: Genomic deletion of chromosome 12p is an independent prognostic marker in prostate cancer

**DOI:** 10.18632/oncotarget.14557

**Published:** 2017-01-09

**Authors:** Martina Kluth, Ramin Ahrary, Claudia Hube-Magg, Malik Ahmed, Heinke Volta, Catina Schwemin, Stefan Steurer, Corinna Wittmer, Waldemar Wilczak, Eike Burandt, Till Krech, Meike Adam, Uwe Michl, Hans Heinzer, Georg Salomon, Markus Graefen, Christina Koop, Sarah Minner, Ronald Simon, Guido Sauter, Thorsten Schlomm

**Affiliations:** ^1^ Institute of Pathology, University Medical Center Hamburg-Eppendorf, Germany; ^2^ Martini-Klinik, Prostate Cancer Center, University Medical Center Hamburg-Eppendorf, Germany; ^3^ Dept. of Urology, Section for translational prostate cancer research, University Medical Center Hamburg-Eppendorf, Germany

**Present**: Due to a production error, proper equal contribution credit for the first and second authors was erroneously eremoved from the published version.

Corrected: The proper contributions are listed below. The publisher sincerely apologizes for this oversight.

Original article: Oncotarget. 2015; 6(29):27966-79. doi: 10.18632/oncotarget.4626.

